# Supplementary material for: The complete chloroplast genome of Colobanthus apetalus (Labill.) Druce: genome organization and comparison with related species
Source: PeerJ. 2018 May 23;6:e4723. doi: 10.7717/peerj.4723 (PMC5970550; doi:10.7717/peerj.4723)
Supplement: Table S1 [file peerj-06-4723-s001.docx]

**List of repeated sequences in the chloroplast genome of *Colobanthus apetalus*.**

| **Repeat length (bp)** | **Strat site of repeat A** | **Repeat A location** | **Repeat A region** | **Strat site of repeat B** | **Repeat B location** | **Repeat B region** | **Repeat type** |
| --- | --- | --- | --- | --- | --- | --- | --- |
| 30 | 26831 | *ndhA* | SSC | 26842 | *ndhA* | SSC | R |
| 30 | 52304 | *ycf2* | IR | 52346 | *ycf2* | IR | F |
| 30 | 52306 | *ycf2* | IR | 52324 | *ycf2* | IR | F |
| 30 | 66791 | IGS (*psbI-trnS-GCU*) | LSC | 93490 | IGS (*psbC-trnS-UGA*) | LSC | F |
| 30 | 66791 | IGS (*psbI-trnS-GCU*) | LSC | 103043 | *trnS-GGA* | LSC | P |
| 30 | 74582 | IGS (*rps2-rpoC2*) | LSC | 74725 | IGS (*rps2-rpoC2*) | LSC | R |
| 30 | 74723 | IGS (*rps2-rpoC2*) | LSC | 89271 | IGS (*trnE-UCC-trnT-GGU*) | LSC | C |
| 30 | 74724 | IGS (*rps2-rpoC2*) | LSC | 89271 | IGS (*trnE-UCC-trnT-GGU*) | LSC | P |
| 30 | 74725 | IGS (*rps2-rpoC2*) | LSC | 89269 | IGS (*trnE-UCC-trnT-GGU*) | LSC | C |
| 30 | 74726 | IGS (*rps2-rpoC2*) | LSC | 89271 | IGS (*trnE-UCC-trnT-GGU*) | LSC | C |
| 30 | 87857 | IGS (*psbM-trnD-GUC*) | LSC | 89273 | IGS (*trnE-UCC-trnT-GGU*) | LSC | R |
| 30 | 87863 | IGS (*psbM-trnD-GUC*) | LSC | 89275 | IGS (*trnE-UCC-trnT-GGU*) | LSC | F |
| 30 | 89054 | IGS (*trnE-UCC-trnT-GGU*) | LSC | 89086 | IGS (*trnE-UCC-trnT-GGU*) | LSC | F |
| 30 | 89271 | IGS (*trnE-UCC-trnT-GGU*) | LSC | 110788 | IGS (*trnM-CAU-atpE*) | LSC | P |
| 30 | 93490 | IGS (*psbC-trnS-UGA*) | LSC | 103043 | *trnS-GGA* | LSC | P |
| 30 | 96652 | *psaB* | LSC | 98876 | *psaA* | LSC | F |
| 30 | 106468 | IGS (*trnF-GAA-ndhJ*) | LSC | 129841 | *clpP1* intron1 | LSC | P |
| 31 | 52327 | *ycf2* | IR | 52351 | *ycf2* | IR | F |
| 31 | 63522 | IGS (*trnK-UUU-rps16*) | LSC | 63528 | IGS (*trnK-UUU-rps16*) | LSC | F |
| 31 | 68412 | *trnG-UCC* | LSC | 94423 | *trnG-GCC* | LSC | F |
| 31 | 89269 | IGS (*trnE-UCC-trnT-GGU*) | LSC | 89269 | IGS (*trnE-UCC-trnT-GGU*) | LSC | R |
| 32 | 89270 | IGS (*trnE-UCC-trnT-GGU*) | LSC | 110784 | IGS (*trnM-CAU-atpE*) | LSC | P |
| 33 | 63522 | IGS (*trnK-UUU-rps16*) | LSC | 63526 | IGS (*trnK-UUU-rps16*) | LSC | F |
| 34 | 89042 | IGS (*trnE-UCC-trnT-GGU*) | LSC | 89074 | IGS (*trnE-UCC-trnT-GGU*) | LSC | F |
| 35 | 63522 | IGS (*trnK-UUU-rps16*) | LSC | 63524 | IGS (*trnK-UUU-rps16*) | LSC | F |
| 38 | 87231 | IGS (*petN-psbM*) | LSC | 87231 | IGS (*petN-psbM*) | LSC | P |
| 40 | 26591 | *ndhA* | SSC | 45868 | IGS (*trnV-GAC-rps7*) | IR | P |
| 41 | 33194 | *ycf1* | SSC | 33194 | *ycf1* | SSC | R |
| 134 | 100444 | IGS (*psaA-ycf3*) | LSC | 118249 | IGS (*psaI-ycf4*) | LSC | P |
| 169 | 100409 | IGS (*psaA-ycf3*) | LSC | 118249 | IGS (*psaI-ycf4*) | LSC | P |

IGS (*psbI-trnS-GCU*) means spacer between *psbI* and *trnS-GCU*, P means palindromic match, F means forward (direct) match, R means reverse match and C means complement match.
