# Supplementary material for: The complete chloroplast genome of Colobanthus apetalus (Labill.) Druce: genome organization and comparison with related species
Source: PeerJ. 2018 May 23;6:e4723. doi: 10.7717/peerj.4723 (PMC5970550; doi:10.7717/peerj.4723)
Supplement: Table S2 [file peerj-06-4723-s002.docx]

**List of repeated sequences in the chloroplast genome of *Colobanthus quitensis***

| **Repeat length (bp)** | **Strat site of repeat A** | **Repeat A location** | **Repeat A region** | **Strat site of repeat B** | **Repeat B location** | **Repeat B region** | **Repeat type** |
| --- | --- | --- | --- | --- | --- | --- | --- |
| 30 | 7 895 | IGS (*psbI-trnS-GCU*) | LSC | 44 096 | *trnS-GGA* | LSC | P |
| 30 | 37 702 | *psaB* | LSC | 39 926 | *psaA* | LSC | F |
| 30 | 7 895 | IGS (*psbI-trnS-GCU*) | LSC | 34 538 | IGS (*psbC-trnS-UGA*) | LSC | F |
| 30 | 15 816 | IGS (*rps2-rpoC2*) | LSC | 47 101 | IGS (*trnL-UAA-trnF-GAA*) | LSC | R |
| 30 | 34 538 | *trnS-UGA* | LSC | 44 096 | *trnS-GGA* | LSC | P |
| 30 | 90 031 | *ycf2* | IR | 90 073 | *ycf2* | IR | F |
| 30 | 90 053 | *ycf2* | IR | 90 071 | *ycf2* | IR | F |
| 31 | 506 | *trnG-UCC* | LSC | 35 473 | *trnG-GCC* | LSC | F |
| 31 | 30 324 | IGS (*trnE-UCC-trnT-GGU*) | LSC | 63 087 | IGS (*petA-psbJ*) | LSC | P |
| 31 | 90 025 | *ycf2* | IR | 90 049 | *ycf2* | IR | F |
| 38 | 28 323 | IGS (*petN-psbM*) | LSC | 28 323 | IGS (*petN-psbM*) | LSC | P |
| 40 | 96 499 | IGS (*rps12-trnV-GAC*) | IR | 118 931 | *ndhA* | SSC | F |
| 41 | 125 521 | *ycf1* | SSC | 125 521 | *ycf1* | SSC | R |
| 134 | 41 494 | IGS (*psaA-ycf3*) | LSC | 59 331 | IGS (*psaI-ycf4*) | LSC | P |
| 165 | 41 463 | IGS (*psaA-ycf3*) | LSC | 59 331 | IGS (*psaI-ycf4*) | LSC | P |

IGS (*psbI-trnS-GCU*) means spacer between *psbI* and *trnS-GCU*, P means palindromic match, F means forward (direst) match and R means reverse match
