# Supplementary material for: The complete chloroplast genome of Colobanthus apetalus (Labill.) Druce: genome organization and comparison with related species
Source: PeerJ. 2018 May 23;6:e4723. doi: 10.7717/peerj.4723 (PMC5970550; doi:10.7717/peerj.4723)
Supplement: Table S3 [file peerj-06-4723-s003.docx]

## Distribution of SSR in the *Colobanthus apetalus* cp genome.

| Type | Repeat unit | Length (bp) | Start | End | Location |
| --- | --- | --- | --- | --- | --- |
| Mononucleotide | A | 13 | 16 448 | 16 460 | *ndhF* |
|  |  | 12 | 31 113 | 31 124 | *ycf1* |
|  |  | 12 | 60 845 | 60 856 | *trnK-UUU* (intron) |
|  |  | 15 | 63 545 | 63 559 | IGS (*trnK-UUU-rps16*) |
|  |  | 12 | 65 851 | 65 862 | IGS (*trnQ-UUG-psbK*) |
|  |  | 17 | 65 897 | 65 913 | IGS( *trnQ-UUG-psbK*) |
|  |  | 12 | 66 488 | 66 499 | *IGS* (*psbK-psbI*) |
|  |  | 19 | 74 593 | 74 611 | IGS (*rps2-rpoC2*) |
|  |  | 19 | 74 727 | 74 745 | IGS (*rps2-rpoC2*) |
|  |  | 12 | 76 803 | 76 814 | *rpoC2* |
|  |  | 24 | 87 864 | 87 887 | IGS (*psbM-trnD-GUC*) |
|  |  | 23 | 89 271 | 89 293 | IGS (*trnE-UUC-trnT-GGU*) |
|  |  | 13 | 90 707 | 90 719 | IGS (*trnT-GGU-psbD*) |
|  |  | 16 | 106 471 | 106 486 | IGS (*trnF-GAA-ndhJ*) |
|  |  | 13 | 110 797 | 110 809 | IGS (*trnM-CAU-atpE*) |
|  |  | 17 | 115 092 | 115 108 | IGS (*rbcL-accD*) |
|  |  | 17 | 124 053 | 124 069 | IGS (*psbE-petL*) |
|  |  | 12 | 128 062 | 128 073 | IGS (*rpl20-rps12*) |
|  |  | 21 | 129 846 | 129 866 | *clpP1* (intron) |
|  |  | 14 | 133 876 | 133 889 | *petB* (intron) |
| Dinucleotide | AT | 23 | 63 523 | 63 545 | IGS (*trnK-UUU-rps16*) |
|  |  | 15 | 66 656 | 66 670 | IGS (*psbI-trnS-GCU*) |
|  |  | 12 | 117 512 | 117 523 | IGS (*accD-psaI*) |
| Trinucleotide | AAT | 13 | 104 019 | 104 031 | IGS (*rps4-trnT-UGU*) |
|  |  | 12 | 20 528 | 20 539 | IGS (*trnL-UAG-ccsA*) |
|  |  | 12 | 137 422 | 137 433 | IGS (*rpoA-rps11*) |
| Tetranucleotide | ACCT | 14 | 38,510 | 38,523 | *rrn23* |
|  | AAGG | 13 | 71,808 | 71,82 | IGS (*atpF-atpH*) |
|  | AAAC | 13 | 94,188 | 94,200 | IGS (*psbZ-trnG-GCC*) |
|  | AACT | 13 | 100,363 | 100,375 | IGS (*psaA-ycf3*) |
|  | AAAT | 12 | 18,722 | 18,733 | IGS (*ndhF-rpl32*) |
|  | AATT | 12 | 27,317 | 27,328 | *ndhA* (intron) |
|  | AATT | 12 | 63,761 | 63,772 | IGS (*trnK-UUU-rps16*) |
|  | AAAC | 12 | 68,894 | 68,905 | *atpA* |
|  | AAAT | 12 | 87,208 | 87,219 | IGS (*petN-psbM*) |
|  | AATT | 12 | 117,836 | 117,847 | IGS (*accD-psaI*) |
|  | AAAG | 12 | 128,845 | 128,856 | *clpP1* (intron) |
|  | AAAT | 12 | 135,290 | 135,301 | *petD* (intron) |
| Pentanucleotide | AAATT | 18 | 19,810 | 19,827 | IGS (*rpl32-trnL-UAG*) |
|  | AAATC | 16 | 24,379 | 24,394 | IGS (*ndhE-ndhG*) |
|  | AATCT | 15 | 123,960 | 123,974 | IGS (*psbE-petL*) |

IGS (*trnK-UUU-rps16*) means spacer between *trnK-UUU* and *rps16*
