# Supplementary material for: The complete chloroplast genome of Colobanthus apetalus (Labill.) Druce: genome organization and comparison with related species
Source: PeerJ. 2018 May 23;6:e4723. doi: 10.7717/peerj.4723 (PMC5970550; doi:10.7717/peerj.4723)
Supplement: Table S4 [file peerj-06-4723-s004.docx]

## Distribution of SSR in the *Colobanthus quitensis* cp genome.

| Type | Repeat unit | Length (bp) | Start | End | Location |
| --- | --- | --- | --- | --- | --- |
| Mononucleotide | A | 14 | 1 900 | 1 913 | *trnK-UUU* (intron) |
|  |  | 14 | 4 584 | 4 597 | IGS (*trnK-UUU-rps16*) |
|  |  | 16 | 4 664 | 4 679 | IGS (*trnK-UUU-rps16*) |
|  |  | 13 | 7 021 | 7 033 | IGS (*trnQ-UUG-psbK*) |
|  |  | 13 | 7 608 | 7 620 | IGS (*psbK-psbI*) |
|  |  | 12 | 12 243 | 12 254 | *atpF* (intron) |
|  |  | 13 | 15 690 | 15 702 | IGS (*rps2-rpoC2*) |
|  |  | 15 | 15 818 | 15 832 | IGS (*rps2-rpoC2*) |
|  |  | 12 | 17 890 | 17 901 | *rpoC2* |
|  |  | 12 | 29 127 | 29 138 | IGS (*psbM-trnD-GUC*) |
|  |  | 16 | 30 325 | 30 340 | IGS (*trnE-UUC-trnT-GGU*) |
|  |  | 15 | 34 385 | 34 399 | IGS (*psbC-trnS-UGA*) |
|  |  | 15 | 45 697 | 45 711 | IGS (*trnT-UGU-trnL-UAA*) |
|  |  | 16 | 47 530 | 47 545 | IGS (*trnF-GAA-ndhJ*) |
|  |  | 19 | 51 859 | 51 877 | IGS (*trnM-CAU-atpE*) |
|  |  | 13 | 56 168 | 56 180 | IGS (*rbcL-accD*) |
|  |  | 12 | 60 286 | 60 297 | IGS (*ycf4-cemA*) |
|  |  | 18 | 63 103 | 63 120 | IGS (*petA-psbJ*) |
|  |  | 13 | 64 918 | 64 930 | IGS (*psbE-petL*) |
|  |  | 16 | 65 162 | 65 177 | IGS (*psbE-petL*) |
|  |  | 12 | 69 182 | 69 193 | IGS (*rpl20-rps12*) |
|  |  | 16 | 70 968 | 70 983 | *clpP1* (intron) |
|  |  | 12 | 74 994 | 75 005 | *petB* (intron) |
|  |  | 14 | 106 113 | 106 126 | IGS (*trnR-ACG-trnN-GUU*) |
|  |  | 13 | 108 773 | 108 785 | *ndhF* |
|  |  | 12 | 123 440 | 123 451 | *ycf1* |
| Dinucleotide | AT | 13 | 4 572 | 4 584 | IGS (*trnL-UAG-ccsA*) |
|  |  | 12 | 58 594 | 58 605 | IGS (*trnK-UUU-rps16*) |
|  |  | 15 | 112 918 | 112 932 | IGS (*accD-psaI*) |
| Trinucleotide | AAT | 13 | 45 072 | 45 084 | IGS (*rps4-trnT-UGU*) |
|  |  | 12 | 78 532 | 78 543 | IGS (*rpoA-rps11*) |
|  |  | 12 | 112 853 | 112 864 | IGS (*trnL-UAG-ccsA*) |
| Tetranucleotide | AATT | 12 | 4 886 | 4 897 | IGS (*trnK-UUU-rps16*) |
|  | AATG | 13 | 9 555 | 9 567 | IGS (*trnG-UCC-trnR-UCU*) |
|  | AAAC | 12 | 9 990 | 10 001 | *atpA* |
|  | AAGG | 13 | 12 905 | 12 917 | IGS (*atpF-atpH*) |
|  | AAAT | 12 | 28 300 | 28 311 | IGS (*petN-psbM*) |
|  | AAAC | 13 | 35 237 | 35 249 | IGS (*psbZ-trnG-GCC*) |
|  | AACT | 13 | 41 413 | 41 425 | IGS (*psaA-ycf3*) |
|  | AATT | 12 | 58 918 | 58 929 | IGS (*accD-psaI*) |
|  | AAAG | 12 | 69 965 | 69 976 | *clpP1* (intron) |
|  | AAAT | 12 | 76 400 | 76 411 | *petD* (intron) |
|  | ACCT | 14 | 103 885 | 103 898 | *rrn23* |
|  | AAAT | 12 | 111 047 | 111 058 | IGS (*ndhF-rpl32*) |
|  | AATT | 16 | 119 640 | 119 655 | *ndhA* (intron) |
| Pentanucleotide | AATCT | 15 | 65,069 | 65,083 | IGS (*psbE-petL*) |
|  | AAATT | 18 | 112,134 | 112,151 | IGS (*rpl32-trnL-UAG*) |
|  | AAATC | 21 | 116,707 | 116,727 | IGS (*ndhE-ndhG*) |

IGS (*trnK-UUU-rps16*) means spacer between *trnK-UUU* and *rps16*
