# Supplementary material for: The complete chloroplast genome of Colobanthus apetalus (Labill.) Druce: genome organization and comparison with related species
Source: PeerJ. 2018 May 23;6:e4723. doi: 10.7717/peerj.4723 (PMC5970550; doi:10.7717/peerj.4723)
Supplement: Table S5 [file peerj-06-4723-s005.docx]

**SNPs within cp genome of *C. apetalus***

| **Position in genome** | **Localization** | **SNP type** | **Coverage (Read number)** | **Minor allele frequency** | **Major allele frequency** |
| --- | --- | --- | --- | --- | --- |
| 132 736 | IGS | T -> C | 68 | 11.8% | 88.2% |
| 133 684 | IGS | C -> A | 35 | 11.4% | 88.6% |
| 136 359 | IGS | T -> G | 37 | 10.8% | 89.2% |
| 142 224 | IGS | A -> T | 33 | 12.1% | 87.9% |

IGS – intergenic spacer
